# Mice Lacking NKT Cells but with a Complete Complement of CD8^+^ T-Cells Are Not Protected against the Metabolic Abnormalities of Diet-Induced Obesity

**DOI:** 10.1371/journal.pone.0019831

**Published:** 2011-06-03

**Authors:** Benjamin S. Mantell, Maja Stefanovic-Racic, Xiao Yang, Nikolas Dedousis, Ian J. Sipula, Robert M. O'Doherty

**Affiliations:** 1 Division of Endocrinology and Metabolism, Department of Medicine, University of Pittsburgh, Pittsburgh, Pennsylvania, United States of America; 2 Department of Microbiology and Molecular Genetics, University of Pittsburgh, Pittsburgh, Pennsylvania, United States of America; University of Padova, Medical School, Italy

## Abstract

The contribution of natural killer T (NKT) cells to the pathogenesis of metabolic abnormalities of obesity is controversial. While the combined genetic deletion of NKT and CD8^+^ T-cells improves glucose tolerance and reduces inflammation, interpretation of these data have been complicated by the recent observation that the deletion of CD8^+^ T-cells alone reduces obesity-induced inflammation and metabolic dysregulation, leaving the issue of the metabolic effects of NKT cell depletion unresolved. To address this question, CD1d null mice (CD1d^−^/^−^), which lack NKT cells but have a full complement of CD8^+^ T-cells, and littermate wild type controls (WT) on a pure C57BL/6J background were exposed to a high fat diet, and glucose intolerance, insulin resistance, dyslipidemia, inflammation, and obesity were assessed. Food intake (15.5±4.3 vs 15.3±1.8 kcal/mouse/day), weight gain (21.8±1.8 vs 22.8±1.4 g) and fat mass (18.6±1.9 vs 19.5±2.1 g) were similar in CD1d^−^/^−^ and WT, respectively. As would be expected from these data, metabolic rate (3.0±0.1 vs 2.9±0.2 ml O_2_/g/h) and activity (21.6±4.3 vs 18.5±2.6 beam breaks/min) were unchanged by NKT cell depletion. Furthermore, the degree of insulin resistance, glucose intolerance, liver steatosis, and adipose and liver inflammatory marker expression (TNFα, IL-6, IL-10, IFN-γ, MCP-1, MIP1α) induced by high fat feeding in CD1d^−^/^−^ were not different from WT. We conclude that deletion of NKT cells, in the absence of alterations in the CD8^+^ T-cell population, is insufficient to protect against the development of the metabolic abnormalities of diet-induced obesity.

## Introduction

Obesity coincides with a state of chronic, low-grade inflammation that has been implicated in the pathogenesis of insulin resistance and dyslipidemia. In particular, it has been demonstrated that macrophages, CD8^+^ T-cells and regulatory T cells in adipose tissue and liver play a role in mediating the detrimental effects of inflammation on metabolism [1]–[6]. However, there continues to be interest in determining the potential role for other immune cells in altering metabolism. In this regard NKT cells are a sub-population of lymphocytes that are proposed to serve as a link between the adaptive and innate immune systems [7]–[9]. NKT cells have receptor expression characteristics of NK cells (they are NK1.1^+^) and T-cells (they express a T-cell receptor (TCR)). Notably, the NKT TCR, rather then being activated by peptide antigens, is activated by glycolipid antigens through the MHC class I-like molecule, CD1d [7]–[9], while an alternative activation pathway is dependent on cytokine signaling from activated dendritic cells [8]. Irrespective of the method, activation of NKT cells results in rapid cytokine production (within hours), which may be of mixed, T_H_1 or T_H_2 dominance depending on the microenvironment to which the NKT cells are exposed and/or different functional subsets of NKT cells [9].

The potential role of NKT cells in altering metabolism has received attention, but the data are inconclusive and somewhat contradictory. Of relevance to the current study are the observations that diet-induced obese mice [10], [11] and *ob/ob* mice [12] have a larger proportion of T_H_1 polarized liver NKT cells [10], and that administration of the NKT activator α-Galactosylceramide to DIO mice exacerbates glucose intolerance and adipose tissue inflammation [13], suggesting that NKT may play a pathogenic role in the metabolic abnormalities associated with these models. Conversely, obesity reduces overall NKT cell numbers in liver [10], [12], [14], while an agonist of NKT cells, glucocerebroside [15], or adoptive transfer of NKT cells [16] improves liver steatosis and glucose tolerance in *ob/ob* mice, suggesting a protective effect of NKT cells. Clearly, an approach that would clarify some of these issues would be to ablate NKT cells and address the consequences of this manipulation on the development of metabolic dysregulation in obesity. Indeed, a recent study [13] demonstrated that mice lacking NKT cells have reduced adipose tissue inflammation and improved glucose tolerance compared to wild-type mice when exposed to a high fat diet. However, the animals used in this study were also depleted of CD8^+^ T-cells, a vitally important caveat, since a recent study demonstrates a role for CD8^+^ T-cells in driving obesity-related inflammation and dysregulated glucose homeostasis [17]. Thus, the metabolic and inflammatory effects of NKT cell deletion alone remain unknown. The current study was undertaken to address this issue. To accomplish this goal, mice lacking CD1d were used. These animals are depleted of NKT cells but maintain a normal complement of CD8^+^ T-cells [18], [19], making it possible to demarcate the effects of NKT cell deletion alone from the effects of combined NKT/CD8^+^ T-cell deletion.

## Materials and Methods

### Animal Care and Maintenance

CD1d^−/−^ mice on a Balb/c background (C.129S2-*Cd1^tm1Gru^*/J) were obtained from Jackson Laboratories. Homozygous mutant mice are deficient in both the *Cd1d1* and *Cd1d2* genes and as a result lack the natural killer T subset[18], [19]. These mice were bred with C57Bl/6 mice to obtain N1F1 heterozygotes, which were subsequently backcrossed to pure C57Bl/6 mice. The gender of the mice in the backcross was alternated with each generation. CD1d heterozygotes of at least the N7 generation (>99% Bl/6 background) were mated to obtain CD1d^−/−^ and wild type littermate controls. Prior to experiments, mice were maintained on a constant 12-h light:12-h dark cycle with free access to water and *ad libitum* fed with a standard chow diet. All procedures were approved by the Institutional Animal Care and Use Committee (IACUC) of the University of Pittsburgh, and were in accordance with the National Research Council's *Guide for the Care and Use of Laboratory Animals.*

### Experimental Design

CD1d^−/−^ and wild type littermate control mice were fed *ad libitum* with a standard chow (SC) or high fat (HF) diet (44% calories from fat – 19% Lard, 1% Corn Oil) from Harlan Teklad for 26 weeks. Mice and food were weighed weekly over the course of the experiment. During this period, mice underwent an analysis of insulin sensitivity using glucose tolerance and insulin tolerance tests, metabolic rate using the CLAMS (Comprehensive Laboratory Animal Monitoring System, OH), and body composition using a Lunar PIXImus densitometer (Lunar, Madison, WI). Whole-body scans, with exclusion of the head, were analyzed for fat and lean masses using the manufacturer's software. At the end of this period, blood and tissues were isolated, flash frozen and stored at −80°C until analysis.

### Glucose Tolerance Tests (GTT), Insulin Tolerance Tests (ITT), and Pyruvate Tolerance Tests (PTT)

For the GTT, 6-hour fasted mice were injected i.p. with 1.5 g/kg glucose. Blood was sampled from the tail vein every 15 minutes for 2 hours post-injection and glucose was measured using an Ascensia Elite glucometer (Bayer, Mishawaka, IN). For the ITT, non-fasted animals were injected i.p. with 1 unit/kg of human recombinant insulin (Humulin R, Lilly, Cincinnati, MO). Blood was sampled from the tail vein every 15 minutes for 2 hours post-injection and glucose was measured using an Ascensia Elite glucometer. For the PTT, 6-hour fasted mice were injected i.p. with 2 g/kg Sodium Pyruvate. Blood was sampled from the tail vein every 15 minutes for 2 hours post-injection and glucose was measured using an Ascensia Elite glucometer (Bayer, Mishawaka, IN).

### Flow Cytometry

Cell surface staining was performed in PBS containing 2% FBS with the following antibodies: anti-CD3-FITC, anti-CD3-PB, anti-CD69-PE, anti-CD8-PerCP, and anti-NK1.1-PE/Cy7. Stained cells were analyzed on an LSR II (BD Biosciences) using FACSDiva software (BD Biosciences).

### Tissue and plasma measurements

Liver triglycerides were determined as previously described [20], [21]. Plasma insulin levels were measured using a commercial kit (by ELISA; ALPCO, Salem, NH) according to the protocol provided by the manufacturer.

### Quantitative reverse transcription-polymerase chain reaction (qRT-PCR)

For analysis of gene expression, total RNA was isolated from liver or adipose tissue using TRIzol Reagent (Invitrogen, Carlsbad, CA) according to the manufacturer's instructions. RT-PCR was carried out using gene specific primers, SYBR green master mix (Bio-Rad, Hercules, CA) and an Applied Biosystems Prism 7300 Real-Time PCR System as previously described [22]. Fold change in mRNA expression was determined using the ΔΔcT method, with all genes normalized to β-actin.

### Statistical Analysis

Data are expressed as means ± SE. Statistical significance was determined by *t*-test and, where appropriate, analysis of variance (repeated measures or one-way ANOVA; Bonferroni's post-hoc test) was performed using the PASW Statistics program (Chicago, IL). Statistical significance was assumed at *P*<0.05.

## Results

### Early and reversible decrease in liver NKT cells in response to a high fat diet

**Figure 1:**
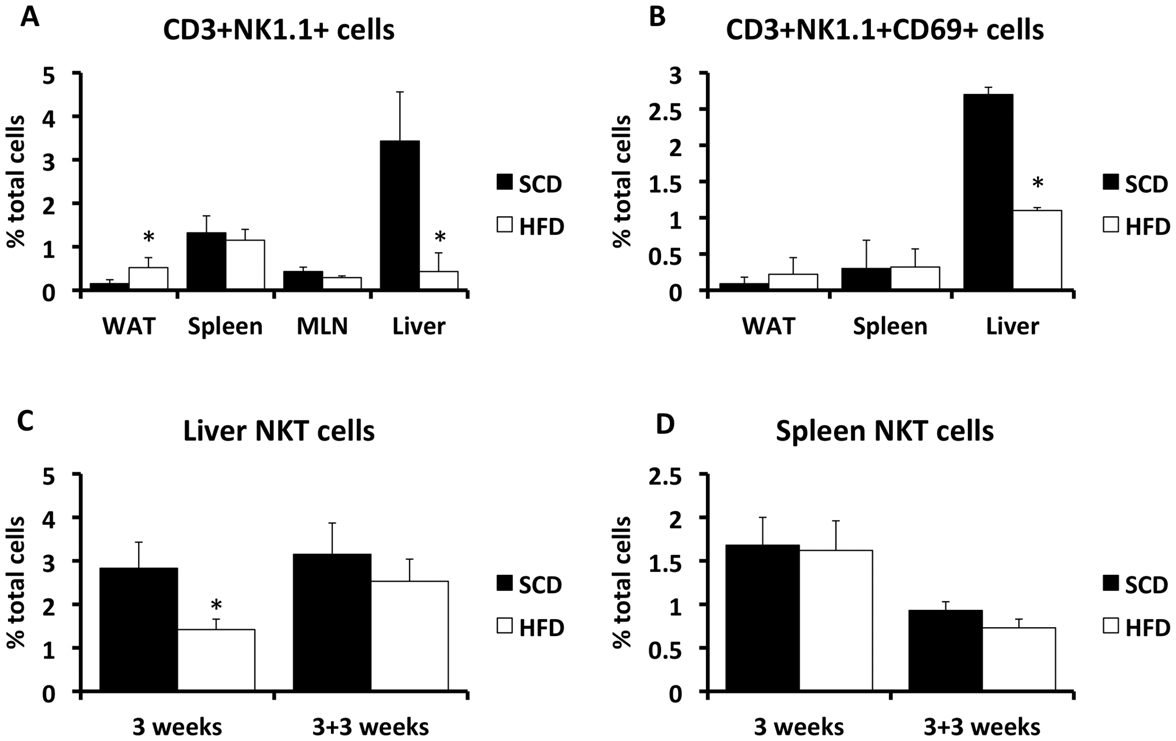
Alterations in tissue NKT-cell composition in the setting of high fat feeding. Wild-type C57Bl/6 mice were placed on a SCD or HFD for 26 weeks (Panels A and B), 3-weeks (Panel C and D), or 3 weeks followed by a 3-week standard chow diet (Panel C and D). White adipose tissue (WAT), spleen, mesenteric lymph nodes (MLN, only for the 26 week study) and liver were collected from lean and obese mice, immune cells were isolated and underwent FACS, as described in Methods. Results are presented as the means±SE for a minimum of 5 animals in each group. Statistical significance is indicated (*).

Previous studies have reported decreases in liver NKT cells in response to a high fat diet. We first sought to confirm and extend these observations, and to assess the effects of diet in other relevant tissues of C57BL/6J mice. In agreement with previous studies [10], [12], [14], there was a selective decrease in the NKT cell population and the NKT activation marker, CD69 in the liver (Figure 1A&B) after exposure to a high fat diet. Conversely, the NKT cell population was enriched in adipose tissue (Figure 1A). NKT populations were not altered in the spleen or mesenteric lymph nodes (MLN). To assess the time-course and reversibility of high fat diet-induced alterations in NKT cells mice were placed on a high fat diet for 3 weeks, and then a proportion of these mice were placed back on a standard chow diet for a further 3 weeks. In response to a 3-week high fat diet, the proportion of NKT cells was reduced to a similar extent as that observed with a prolonged diet. Furthermore, reversion to a standard chow diet for 3 weeks was sufficient to reverse the alterations in NKT cells (Figure 1C). Additionally, there was no change in NKT cell numbers in the spleen at 3 weeks of HFD or when subsequently placed on a standard chow diet for a further 3 weeks (Figure 1D).

### NKT depletion does not alter weight gain, food intake, adiposity, or energy expenditure

**Figure 2:**
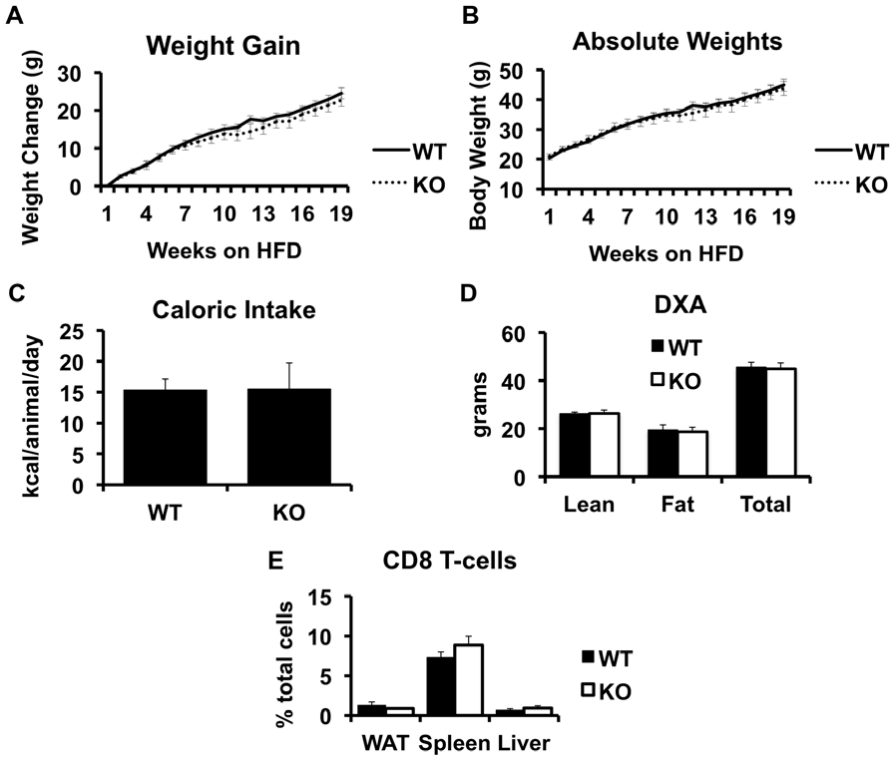
Weight gain and body composition of wild type (WT) and CD1d null (KO) littermate mice on a high fat diet. Weight gain and caloric intake were assessed in high-fat fed wild-type and CD1d knock out mice, as described in Methods (Panels A–C). All mice underwent dual x-ray absorbitometry (DXA) as described in Methods (Panel D). CD8^+^ T-cells were assessed by FACS (Panel E). Results are presented as the means±SE for a minimum of 5 animals in each group.

**Figure 3:**
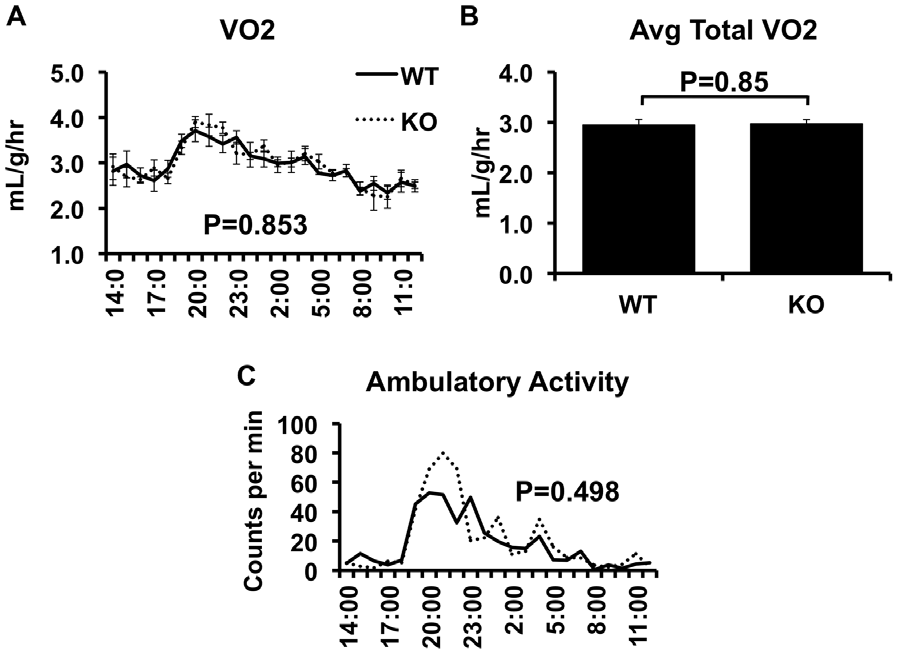
CLAMS analysis. High fat fed wild-type (WT) and CD1d null (KO) mice underwent CLAMS analysis as described in Methods. Data for VO_2_ (Panels A and B), and ambulatory activity (Panel C) are presented. Results are presented as the means±SE for 4 animals in each group.

The CD1d^−/−^ null mouse lacks NKT cells but has a normal complement of CD8^+^ T-cells ([18], [19] and Figure 2E). CD1d^−/−^ mice and littermate wild-type controls (WT), on a pure C57BL/6J background, were exposed to a high fat diet, and a number of metabolic variables were assessed. Weight gain, caloric intake, and body composition were unaffected by NKT cell deletion (Figure 2A–D). Furthermore, indirect calorimetry demonstrated that metabolic rate and activity were similar in CD1d^−/−^ and WT mice (Figure 3A–C), as would be expected given the lack of an effect on NKT depletion on body composition and caloric intake.

### Insulin sensitivity and glucose tolerance are unaffected by NKT cell deletion

**Figure 4:**
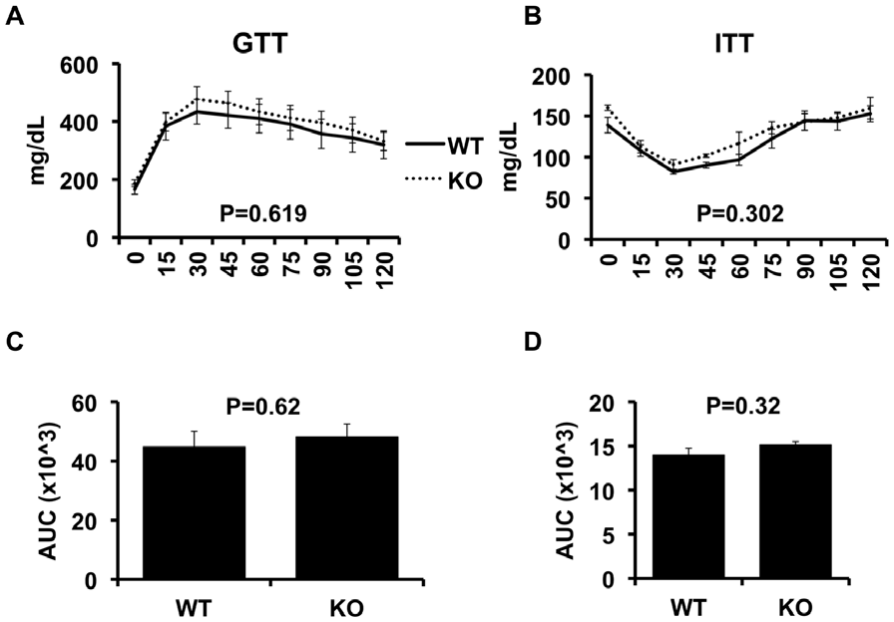
Glucose and insulin tolerance in wild-type and CD1d null mice. High fat fed wild-type (WT, n = 11) and CD1d null (KO, n = 9) mice underwent glucose tolerance tests (GTT) as described in Methods. After 1 week for recovery, all mice underwent insulin tolerance tests (ITT) as described in Methods. Results are presented as the means±SE.

**Figure 5:**
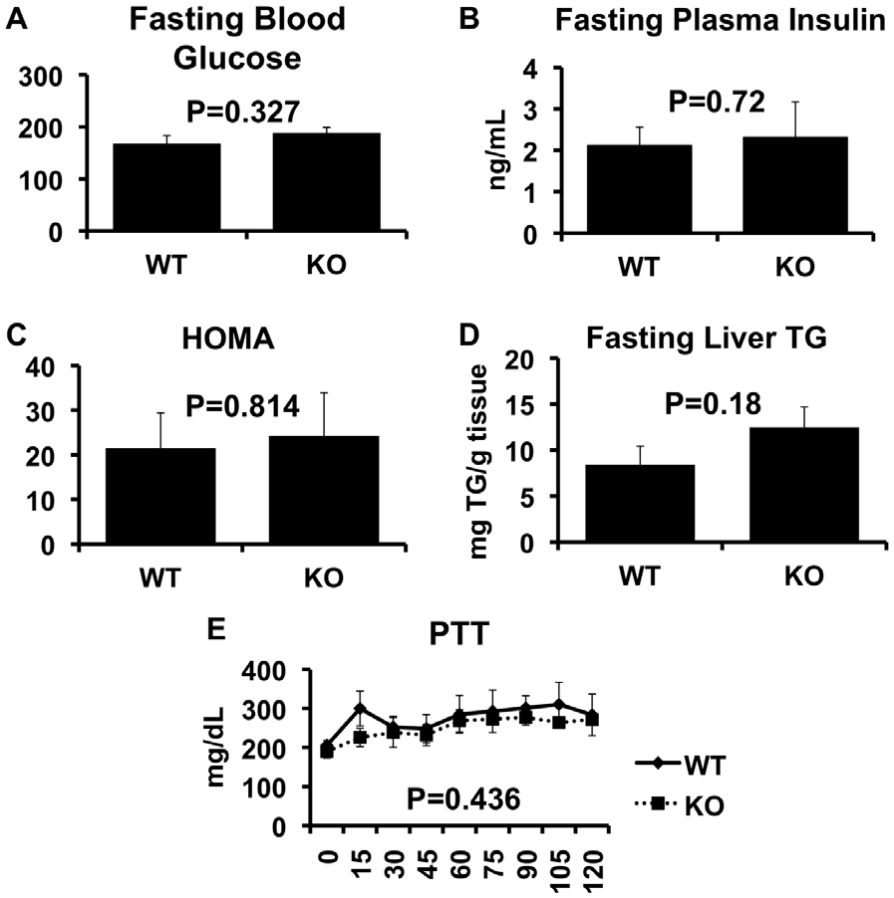
HOMA, Hepatic Glucose Output and liver triglycerides in wild-type and CD1d null mice. Fasting blood glucose (Panel A, n = 11 WT and n = 9 KO) and plasma insulin (Panel B, n = 7 WT, n = 5 KO) concentrations, Pyruvate Tolerance Test (Panel E, n = 4 WT and n = 4 KO) and liver triglycerides (Panel D, n = 11 WT and n = 9 KO) were assessed in WT and KO mice as described in Methods. Results are presented as the means±SE.

We next assessed the effects of NKT deletion on obesity-induced insulin resistance (Figure 4), using insulin (ITT) and glucose tolerance tests (GTT). As Figure 4, Panels A&B show, the GTT and ITT demonstrate that the degree of insulin resistance in obese CD1d^−/−^ mice was similar to obese WT mice. This is also indicated by the area under the curve for the GTT and ITT (Figure 4C and 4D, respectively). Fasting blood glucose concentrations were similar between WT and CD1d^−/−^ mice, as were the fasting insulin concentrations and HOMA values (Figure 5A, B&C). Liver triglyceride levels were also measured and again, no difference between the groups was observed (Figure 5D). Finally, Pyruvate Tolerance Test (Figure 5E) indicated no difference between the two groups.

### Deletion of NKT cells does not alter expression of inflammatory markers

**Figure 6:**
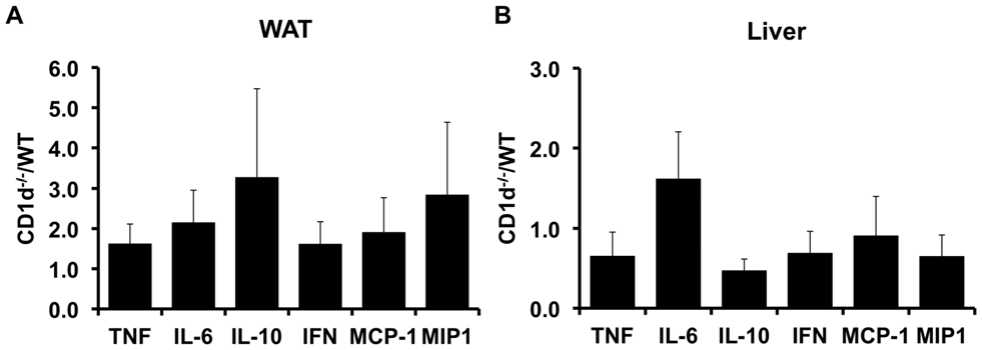
Inflammatory marker expression in adipose tissue and liver of wild-type and CD1d null mice. qRT-PCR was used to assess the expression of indicated genes in liver and adipose tissue from high fat fed wild-type (WT) and CD1d null (KO) mice. Results are presented as ΔΔCt (KO/WT) ±SE for a minimum of 5 animals in each group.

The inflammatory response in obesity is well described and involves increased expression of T_H_1 cytokines and macrophage infiltration/activation into adipose tissue and liver[1], [3], [4], [23], [24]. A previous study[13] demonstrated that the combined deletion of CD8^+^ T-cells and NKT cells reduced adipose tissue inflammation in obesity. However, the effects of NKT deletion alone are unknown. Thus, we next assessed the effects of NKT deletion on the expression of inflammatory markers in WAT and liver of obese CD1d^−/−^ and WT mice. As figure 6 shows, qRT-PCR analysis of TNFα, IL-6, IL-10, IFN-γ, MCP-1 and MIP1α demonstrated that expression of these genes was similar in CD1d^−/−^ and WT animals. These data, taken with the data presented above demonstrate that deletion of NKT cells alone is not sufficient to alter the inflammatory status of obesity.

## Discussion

The major goal of this study was to address the contribution of NKT cells to the development of the metabolic abnormalities of obesity, motivated by previous reports that have suggested metabolic and inflammatory effects of altered NKT activity. These studies have either indirectly inferred roles for NKT cells[10], [12], [14], altered the activity/numbers of NKT cells in pre-existing obesity models[11], [13], [15], [16], [25], or have deleted other cell types i.e. CD8^+^ T-cells, in addition to NKT cells[13], that have been implicated in mediating increased inflammation and metabolic dysregulation in obesity. Importantly, no previous studies have been undertaken in models with a specific deletion of NKT cells.

To investigate the role of NKT cells in the development of obesity-related metabolic disturbances in the absence of alterations in CD8^+^ T-cells, we utilized the CD1d knockout mouse line, which lacks NKT cells, but has a full complement of CD8^+^ T-cells, as opposed to the β2-microglobulin knockout mouse used in a previous study, which lack both NKT and CD8^+^ T-cells[13]. Furthermore, and again unlike study of Ohmura et al.[13], the current study controlled for the genetic background of the mice by utilizing wild-type littermates of the C57Bl/6J CD1^−/−^ mice. The Ohmura study demonstrated that deletion of both NKT and CD8^+^ T-cells results in an improvement in the glucose intolerance and inflammation resulting from exposure of the animals to a high fat diet. The authors conclude that it is unlikely that CD8^+^ T-cells contribute to this phenotype. However, given the more recent study of Nishimura et al.[17], which demonstrates a role for CD8^+^ T-cells in mediating the inflammation and metabolic abnormalities of obesity, and the data obtained in the current study, a more likely explanation is that much, if not all, of the improvements in metabolic function and inflammation observed in the β2-microglobulin null mouse are due to the absence of CD8^+^ T-cells.

A number of studies have addressed the effects of obesity on NKT cell populations and activity[10], [11], [14] and the effects of interventions that alter NKT cell activity or number[11], [13], [15], [16], [25] on the metabolic abnormalities of obesity. By putting the current observations in the context of these studies, it is possible to conjecture on the various and somewhat contradictory conclusions reached in these reports. The issue of the contribution of CD8^+^ T-cells to the metabolic phenotype of β2-microglobulin null mouse has been dealt with above. Turning to the effects of obesity on NKT cell populations and activity, it is now well established that obesity reduces liver NKT cell numbers[10], [12], [14], and that the remaining NKT cells are T_H_1 polarized[14], [25]. However, although these studies are suggestive and are useful for hypothesis generation, by themselves they do not demonstrate a causative role for NKT cells in development of the metabolic or inflammatory abnormalities of obesity. More direct studies have attempted to manipulate the activity or number of NKT cells in vivo. One approach has been to administer glycolipids, which are NKT cell agonists, to obese animals and determine the metabolic consequences of this intervention. Thus, Ohmura et al. report that administration of α-Galactosylceramide (αGC), which is reported to expand the iNKT population[13], to DIO or *ob/ob* mice exacerbates adipose tissue inflammation (DIO and *ob/ob*) and glucose intolerance (DIO). However, while the effects of αGC on inflammatory status in this study are substantial, the effects on glucose tolerance are small, although they are statistically significant. Administration of glucocerebroside (β-Glucosylceramide) is reported to improve liver steatosis and glucose intolerance in *ob/ob* mice[15], although the effects of this glycolipid on NKT cell activity is unclear. Unfortunately, NKT cell activity was not assessed in this study. However, it is possible that these two glycolipids have differential effects on NKT cell activity or expand different subsets of NKT cells, although these issues have not been addressed. Finally, adoptive transfer of NKT cells improves liver steatosis and glucose tolerance in *ob/ob* mice [16]. Unfortunately, polarization of the transferred cells was not assessed in this study, so again we are left to conjecture that perhaps the beneficial effects are the result of the specific NKT cell subset used in this study and/or their polarization. Putting these studies in the context of the current study, we can say that ablation of NKT cells does not appear to protect against or contribute to the metabolic abnormalities of obesity. However, interventions that alter specific NKT populations or their activity in a pre-existing state of obesity appear to result in alterations in inflammatory status and metabolic function, the direction of which may be dependent on the specific NKT population targeted or the effects of the intervention on NKT polarization.

Two observations worthy of note are the rapidity with which nutrient composition-induced alterations in the liver NKT cell population occurs, and the different responses of the liver and adipose tissue NKT populations in obesity. We demonstrate that as little as 3 weeks of high fat diet exposure is sufficient to induce alterations in the liver NKT population. Furthermore, these changes are rapidly reversed when mice are returned to a low fat chow diet. Together, these data indicate that the liver microenvironment is very sensitive to altered nutrient states, and as for longer term diets, these changes correlate with hepatic metabolic and inflammatory alterations, since it is well-established that liver steatosis and insulin resistance and inflammation are present in rodent models within a 1–3 week exposure to a high fat diet. We analyzed multiple tissues of obese C57Bl/6 mice to extend previous observations of decreases in liver NKT cells. As others have shown, we observed a decrease in the percentage of NKT cells in the liver [10], [25], however we also found an increase in NKT cells in the white adipose tissue. As opposed to Miyazaki et al. [11], but similar to Li et al.[10], we did not see a difference in the NKT populations in the spleen. In conclusion, the current study emphasizes the complex relationship between NKT cells and metabolism and suggests that the simple removal of NKT cells does not influence metabolic regulation in obesity.
